# Being Born in Winter–Spring and at Around the Time of an Influenza Pandemic Are Risk Factors for the Development of Schizophrenia: The Apna Study in Navarre, Spain

**DOI:** 10.3390/jcm10132859

**Published:** 2021-06-28

**Authors:** Miguel A. Alvarez-Mon, Sara Guillen-Aguinaga, Victor Pereira-Sanchez, Luc Onambele, Moad J. Al-Rahamneh, Antonio Brugos-Larumbe, Francisco Guillen-Grima, Felipe Ortuño

**Affiliations:** 1 Department of Psychiatry and Medical Psychology, University of Navarra Clinic, 31008 Pamplona, Spain; vpereira@alumni.unav.es (V.P.-S.); fortunos@unav.es (F.O.); 2 Department of Medicine and Medical Specialities, University of Alcala, 28805 Alcala de Henares, Spain; 3 Primary Health Care, Navarra Health Service, 31008 Pamplona, Spain; sguillen.4@alumni.unav.es (S.G.-A.); ablm649@gmail.com (A.B.-L.); 4 Department of Health Sciences, Public University of Navarra (UPNA), 31008 Pamplona, Spain; frguillen@unav.es; 5 Department of Child and Adolescent Psychiatry, NYU Grossman School of Medicine, New York, NY 10016, USA; 6 École des Sciences de la Santé, Université Catholique d’Afrique Centrale, Yaoundé, Cameroon; onambele_luc@yahoo.fr; 7 The Eastern Mediterranean Public Health Network (EMPHNET), Global Health Development (GHD), Amman 11195, Jordan; moad.rahamneh@gmail.com; 8 Department of Preventive Medicine, University of Navarra Clinic, 31008 Pamplona, Spain; 9 Healthcare Research Institute of Navarre (IdiSNA), 31008 Pamplona, Spain

**Keywords:** season of birth, influenza pandemic, neurodevelopmental hypothesis, schizophrenia

## Abstract

Background: We analyzed the relationship between the prevalence of schizophrenia and the season of birth and gestation during a period of an influenza pandemic. Methods: Cross-sectional analysis of a prospective population-based cohort of 470,942 adults. We fitted multivariant logistic regression models to determine whether the season of birth and birth in an influenza-pandemic year (1957, 1968, 1977) was associated with schizophrenia. Results: 2077 subjects had been diagnosed with schizophrenia. Logistic regression identified a significantly greater prevalence of schizophrenia in men than in women (OR = 1.516, CI 95% = 1.388–1.665); in those born in the winter or spring than in those born in the summer or autumn (OR = 1.112, CI 95% = 1.020–1.212); and in those born in a period of an influenza pandemic (OR = 1.335, CI 95% = 1.199–1.486). The increase in risk was also significant when each influenza pandemic year was analyzed separately. However, neither month of birth nor season of birth, when each of the four were studied individually, were associated with a statistically significant increase in that risk. Conclusions: The winter–spring period and the influenza pandemics are independent risk factors for developing schizophrenia. This study contradicts many previous studies and thus revitalizes a locked debate in understanding the neurodevelopmental hypothesis of this disorder.

## 1. Introduction

Schizophrenia is a neurodevelopmental disorder of complex etiopathogenesis involving genetic predispositions and processes based on acquired characteristics deriving from environmental factors [1,2,3].

The search for environmental factors implicated in the pathogenesis of schizophrenia gave rise to retrospective studies exploring the circumstances surrounding the gestation and birth of patients with the disease. Observational studies fed the suspicion that the season of birth was a risk factor for developing the illness [4,5,6]. However, the published results of studies are heterogeneous in view of different latitudes and socioeconomic settings [7,8,9,10,11,12,13,14].

Another environmental factor that has been implicated in the pathogenesis of schizophrenia is the exposure of the subject’s mother to infectious agents during pregnancy [15,16,17]. The relevance of infection by the influenza virus, which causes recurrent world pandemics, is in dispute [18,19]. Studies in experimental models support the implication that the influenza virus can cause embryonic neurodevelopment damage during pregnancy, such as that described for embryos that later develop schizophrenia [1,20,21]. The results of epidemiological studies of the association between schizophrenia and actual infection or possible infection (for example, if gestation occurred during a documented influenza pandemic) are contradictory. For instance, some authors have found that influenza (H1N1) infection during the first trimester of pregnancy was associated with schizophrenia [22], while other authors have found no such association [23,24,25].

One way to resolve the controversy over the role of environmental factors during gestation on the subsequent development of schizophrenia is to conduct large-population epidemiological studies, comparing schizophrenic subjects with those without the disease [26]. If such studies are carried out in populations where everyone has easy access to recognized high-quality health care, the data obtained allows for more precise and consistent results.

The objective of the current study is to analyze, in a large cohort from the north of Spain, the relationship between the prevalence of schizophrenia and the following two factors: (a) season of birth and (b) gestation during a period of an influenza pandemic. The population studied represents a European-Mediterranean population in which citizens have a long life expectancy and easy access to a high-quality health care system.

## 2. Materials and Methods

### 2.1. The Population Studied

A cross-sectional observational study was carried out in a population of 470,942 people over 18 years old in the autonomous community of Navarre (northern Spain). This population is included in a prospective cohort of the Navarre primary health care system (APNA). The cohort was established in 2004; it initially comprised everybody assigned to primary health care at seven primary health centers in the region. In 2012, the cohort was expanded to the whole population of Navarra. The entire population’s health care data since 2004 were incorporated into the database. People enter the cohort at 18 years old. They leave the cohort when they die or emigrate. The data analyzed in this study are cross-sectional from 2012 [27,28,29,30].

The cohort database includes data on the birth date and sex of each person. For the current study, patients with schizophrenia were identified in the database by means of a previous diagnosis of the illness made by Navarre health service doctors specialized in psychiatry. Diagnoses were made based on ICD-10. For the study, subjects who had not been diagnosed with schizophrenia were considered free from the illness.

The season of birth was categorized into summer–autumn (from the beginning of June until the end of November) or winter–spring (from the beginning of December to May). We chose this categorization because previous studies have found that being born in the winter or spring is associated with a higher risk of schizophrenia [4,5,8,31]. In addition, we analyzed the relevance of the season of birth, dividing subjects into the four categories corresponding to each of the four seasons: summer (June, July, August), autumn (September, October, November), winter (December, January, February), and spring (March, April, and May); the subjects analyzed were also grouped by month of birth.

The relevance of exposure to the influenza virus during gestation was studied by grouping subjects according to whether their birth dates occurred within or around periods of influenza pandemics in the second half of the 20th century. The years identified were 1957, 1968, and 1977. The dates for these groups were from January of the year of the pandemic (as given above) until December of the subsequent year. Note that because the cohort only includes people over 18 years of age, data for patients born during the influenza A subtype H1N1 outbreak of 2009 were not eligible.

### 2.2. Statistical Analysis

Univariate tests were used for the comparison of groups in the descriptive analysis of the sample. Variables studied were age, sex, diagnosis of schizophrenia, the season of birth, and birth during (or gestation around the time of) an influenza pandemic. Univariate tests were applied to all the possible combinations of the above variables.

**Figure 1:**
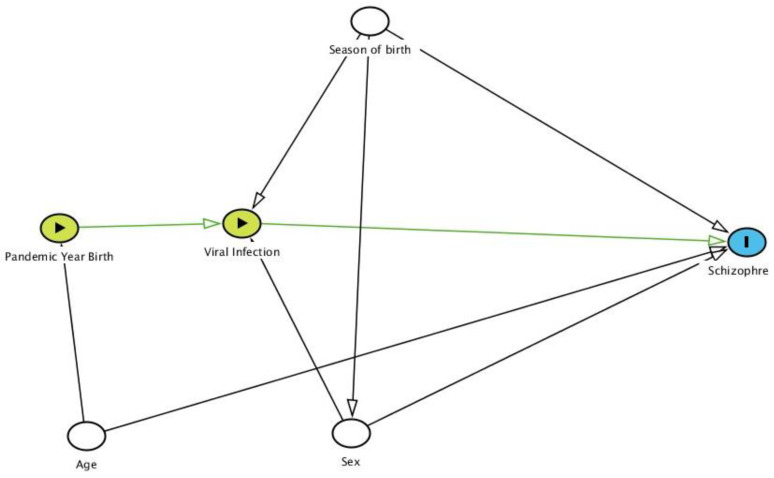
Directed Acyclic Graph (DAG) of the relationship of the season of birth and birth during an Influenza Pandemic, in combination with a viral infection during pregnancy (exposure), with schizophrenia (outcome). Adjusted variables (white circle). Based on DAGitty version 3.0 [32].

Using the Directed Acyclic Graphic (DAG) (Figure 1) [32], we studied the relationship of birth in an influenza pandemic year with schizophrenia prevalence. We detected the following as potential confounding factors: season of birth, age, and sex.

To better study interactions between possible risk factors, multivariate logistic regression was used to analyze differences in the prevalence of schizophrenia for the variables mentioned above. Results were expressed as absolute values and prevalence odds ratios (OR) with 95% confidence intervals. Directed Acyclic Graph (DAG) was computed with DAGitty version 3.0 [32]. Data were analyzed using IBM SPSS Statistics version 20.

## 3. Results

**Figure 2:**
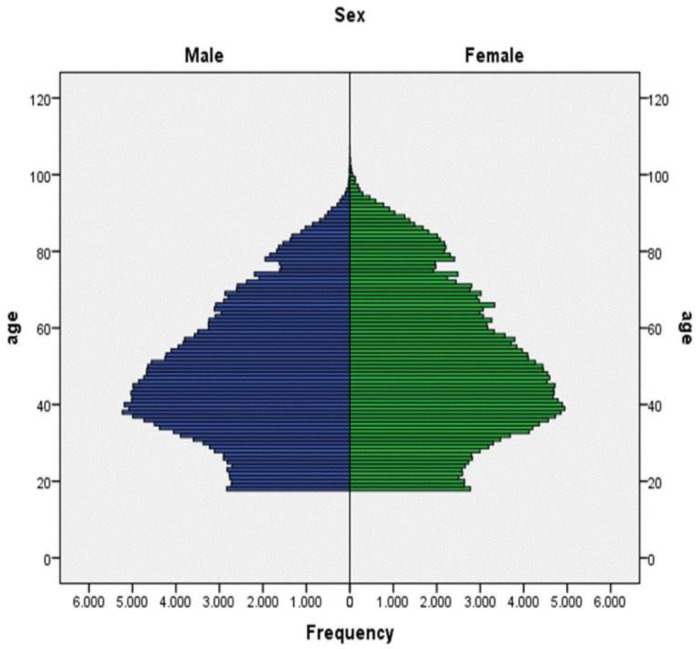
Age structure of the population.

Figure 2 shows the population’s age structure, and Table 1 provides socio-demographic data on the population sample. Out of 470,942 subjects, 2077 had been previously diagnosed with schizophrenia, which is a prevalence of 0.44%. The whole population was divided into the following age groups: individuals up to thirty years old; followed by those between 30 and 100, grouped by decades; and finally, those over 100 years old.

The prevalence of schizophrenia by age group and sex is shown in Table 2. The crude prevalence odds ratios and the adjusted odds ratios determined by logistic regression are given in Table 3. Both crude and adjusted odds ratios indicate a significant increase in the prevalence of schizophrenia in males relative to that in females (OR = 1.516, 95% CI: 1.388–1.655) (in older subjects, the relationship is less marked), in persons born in winter–spring relative to those born in summer–autumn (OR = 1.112, 95% CI: 1.020–1.212), and in subjects born during (or the year after) years of an influenza pandemic relative to subjects born in non-pandemic years (OR = 1.349, 95% CI = 1.208–1.507). The latter relationship held true for each pandemic studied independently of the others, with ORs of 1.476 (95% CI: 1.244–1.750) for the 1957–1959 pandemic, 1.261 (95% CI: 1.060–1.493) for the 1968–1970 pandemic, and 1.280 (95% CI: 1072–1528) for the 1977–1978 pandemic. There were no findings that revealed an interaction between the variables studied. No statistically significant associations were found between schizophrenia and either individual month of birth or individual season of birth.

## 4. Discussion

The study provides additional evidence concerning the effect of environmental factors during the perinatal period on the risk of developing schizophrenia. Our research on a large population that is representative of a community with a high economic level and access to a fully developed public health care system found a significant increase in the risk of schizophrenia for subjects born in the winter–spring period and who were under gestation or born during the influenza pandemics of 1957, 1968, and 1977. We did not find any interaction between the factors of the season of birth and the influenza pandemic years. The prevalence of schizophrenia in the population was higher for males than females.

Based on research findings, it has been postulated that the season of the year in which a person is born affects the risk of developing schizophrenia. However, other findings contradict this [33]. Interestingly, the month of birth has also been associated with different important health outcomes such as the risk of suicide or lifespan [34,35]. Moreover, it has been proposed that the latitude and climate of the regions studied complicate the comparison of results. Thus, there is a more remarkable similarity between results obtained from populations in the northern hemisphere than between results from populations in tropical regions [10,36]. Additionally, meteorological parameters such as temperature, humidity, and maternal exposure to the sun during pregnancy have been implicated as causes of variability in results [37].

The current study is a cross-sectional analysis of a prospective cohort that began with a range of primary healthcare centers and expanded to include practically the whole population of the autonomous community of Navarre in northern Spain [27,28,29,30]. It should be noted that the population studied is subject to a clear cycle of four seasons in the year. Our results show that being born in winter–spring increases the risk of suffering schizophrenia relative to being born in summer–autumn. Therefore, our study confirms the “seasonality of schizophrenia’’. In Navarre, the season with the least hours of sun is winter, so lower levels of vitamin D might be expected during this time. Furthermore, vitamin D deficiency during pregnancy has been implicated in the increased the risk of the offspring developing schizophrenia [38]. Diet during pregnancy has been postulated as a risk factor [39,40]. In this respect, it may be relevant to mention that the predominant diet of the population analyzed is the so-called Mediterranean diet [41,42].

Among possible risk factors for developing schizophrenia are maternal infections during pregnancy [15,16,17]. This effect has been related to an interference in neurodevelopment, caused by inflammation that was induced by infectious agents [21]. One of the infectious agents investigated in relation to schizophrenia is the influenza virus. Results in experimental models indicate that intrauterine infection by means of a virus provokes alterations in the neurodevelopment of the embryo, and that these alterations are consistent with schizophrenia [1,20,21]. However, the results of epidemiological association studies are contradictory, and without a consistent demonstration of increased risk, the scientific debate in this area remains open [22,23,24,25]. Two meta-analysis studies concluded that there was no significant association between birth during the 1957 pandemic and schizophrenia [18,19]. However, a qualitative review of studies on the risk of schizophrenia in relation to various types of infection—but with particular attention to influenza infection in the 20th century –concluded that maternal infection with the influenza virus during pregnancy was a risk factor for the development of schizophrenia in the offspring [2].

Most studies assume that all people born during an influenza pandemic were exposed to the virus–the ecological assumption. However, there are other studies in which individuals exposed to the virus during the various date ranges are identified based on information from the parents or data from serological analyses of samples from the mother during pregnancy. The studies included in the three meta-analyses and the qualitative review referred to above are heterogeneous regarding epidemiological criteria and the size of the population sample [16,18,19,43,44]. Our study, which used an individual approach for date of birth and clinical diagnosis, and an ecological approach for exposure to the virus during pandemics, found a clear statistical relationship between risk and birth during or after the influenza pandemics of 1957, 1968, and 1977. The association was not related to the season of birth, age, and sex, and held true for each pandemic independently of the others. The population sample was large in comparison with many previous studies. Our results contradict an earlier study concerning the 1957 pandemic [19] and re-establish the validity of asking whether influenza infection during gestation is a risk factor for schizophrenia.

The differences between our results and those of other studies cited in recent reviews might be due to methodological considerations or to unknown differences in the characteristics of our sample. It should be noted that for the three pandemics studied, we included subjects born in the year of the pandemic and the subsequent year. This strategy ensures the inclusion of all subjects exposed to the virus irrespective of their gestational or neonatal age during the actual pandemic.

In previous studies, OR or relative risk (RR) were calculated as measures of association. Here, we report OR values from logistic regression. When the frequency of the phenomenon studied is low, RR and OR tend towards equivalence. Therefore, our results can be directly compared to other population studies on schizophrenia, whether they use OR or RR [45].

The neurodevelopment hypothesis for schizophrenia deserves deeper, multimodal research [1]. Like those of other authors, our results highlight the fact that exposure to the influenza virus during gestation is not sufficient or necessary for the development of schizophrenia. Other environmental factors during pregnancy and the first few years of life, in conjunction with genetic vulnerability, most likely determine the final risk [3,46]. In our study, the magnitude of the association between the pandemic and schizophrenia seems to fall from 1957 to 1977. This reduction might be related to improvements in hygiene, nutrition, and the health service that occurred during the second half of the 20th century in the region of the population analyzed.

Studies based on ecological approximations, looking at large populations, and focusing on a single agent or factor are helpful for obtaining preliminary determinations of risk factors. Still, they fail to provide precise information about the complex pathogenesis process in each individual. Thus, laboratory studies and translational research will be needed to understand how the influenza virus and other infectious agents affect early neurological development [3].

Finally, it is important to clarify that the prevalence of schizophrenia in different studies varies [47]. This variability may result from various factors, including genetic and racial, environmental, and socio-cultural factors. In our research, we found that the illness affected 0.44% of the population. This prevalence is at the lower end of the range worldwide [48,49]. Furthermore, our data show that the prevalence of this chronic illness decreased in those over 80 years old. This finding is consistent with the observation that schizophrenia patients have a shorter life expectancy than non-schizophrenia subjects [50,51,52]. In general, the population studied here has a long average life expectancy.

Our study has some limitations. The cross-sectional nature in which clinical data were collected means that we were dealing with prevalence instead of incidence. This suggests the risk of possible diagnosis error. Prevalence is related to survival. Deaths before the date of the study may produce an underestimation of the prevalence. However, given that our sample was highly representative and given the longevity of our population, it is improbable that any such missed subjects would show important differences relative to those that remained alive. It is unlikely that exposure of those people to the influenza virus during a pandemic would be related to their early death.

In the community studied, medical care in general and psychiatric attention are accessible and universal. In addition, the available retrospective and cross-sectional data are reliable. The ecological assumption of global exposure to the influenza virus during an influenza pandemic is a limitation of the study; however, as pointed out in other studies, ecological studies in this field are valid, given that an influenza pandemic implies exposure of up to 50% of the population [18,19].

Our analysis did not extend to a breakdown of the effect of pandemics by the trimester of pregnancy while a pandemic was in full swing. Instead, we pooled all subjects born within a broad time window around each pandemic period and extended it to include the year after the pandemic. The approach adopted means that we could not obtain results specific to the trimester of gestation. However, it avoids the risk of multiplying the number of statistical comparisons. It also avoids the need to assume that an individual’s exposure to the virus occurred at a specific moment (i.e., the peak period of the pandemic) when, in fact, it could have happened at any time throughout the pandemic. Our data did not permit the investigation of the devastating influenza pandemic of 1918 [53].

Finally, the increased risk that we found is less than double the basal risk. Thus, our results can be criticized as being of low magnitude and therefore, as being spurious. It is worth noting, however, that the results were consistent, both with and without adjustment for confounding factors and that our findings concerning pandemics were consistent for all three pandemics. Furthermore, our findings for the season of birth grouped as winter–spring versus summer–autumn are consistent with published studies in the literature [4,5,8,31]. Finally, the low relative magnitude of associations is consistent with associations in general with risk factors in psychoses, which for the most part have OR/RR values of less than three to date; there are few individual factors of high risk [3]. This only confirms the complexity and multifactorial nature of the etiopathogenesis of schizophrenia [16].

The most relevant strengths of this study are its size and representativeness of a population and the multivariate measurement of the effects of several factors, making it possible to explore potential interactions between factors and carry out an adjusted analysis. The most important clinical implication of the association found between influenza and schizophrenia is that it might be possible to use existing measures against influenza to help prevent schizophrenia.

The results obtained in this research support the hypothesis that environmental factors, especially infections during gestation, increase the risk of development of schizophrenia. Thus, our findings also support the neurodevelopment theory of the etiopathogenesis of the illness. However, we believe that further multimodal research is required to verify the validity of these hypotheses and to explore the role of influenza in developing other severe psychiatric conditions such as bipolar disorder [54]. Such future research should include longitudinal data on the exposure of individual subjects to multiple environmental and genetic risk factors. When supported by basic and translational research, such a focus can answer the question of whether influenza vaccination for pregnant mothers is a useful prophylactic measure for schizophrenia and will provide a better understanding of the neurobiology of schizophrenia and realistic approaches to prevention and early intervention.

## Figures and Tables

**Table 1:** Socio-demographic data of the population.

	Total	
	N (Mean)	% (SD)
Age	5046	18.45
18–30	67,239	14.28%
31–40	86,097	18.28%
41–50	95,766	20.33%
51–60	79,564	16.89%
61–70	61,553	13.07%
71–80	43,776	9.30%
81–90	30,114	6.39%
91–100	6626	1.41%
>100	207	0.04%
Total	470,942	100%
Sex		
Female	238,403	50.6%
Male	232,539	49.4%
Total	470,942	100.0
Schizophrenia		
No	468,865	99.56%
Yes	2077	0.44%
Total	470,942	100.0

**Table 2:** Prevalence of Schizophrenia by age and sex.

	Male		Female		Total	
Age	N	%	N	%	N	%
−25	22,168	0.2%	21,115	0.1%	43,283	0.2%
−30	15,524	0.4%	15,108	0.2%	30,632	0.3%
−35	20,419	0.6%	19,861	0.3%	40,280	0.4%
−40	25,231	0.8%	23,991	0.3%	49,222	0.5%
−45	25,035	0.7%	23,574	0.3%	48,609	0.5%
−50	23,588	0.7%	22,576	0.4%	46,164	0.6%
−55	21,100	0.6%	20,269	0.4%	41,369	0.5%
−60	17,957	0.6%	17,572	0.6%	35,529	0.6%
−65	15,667	0.5%	15,554	0.5%	31,221	0.5%
−70	14,261	0.4%	15,016	0.4%	29,277	0.4%
−75	10,858	0.4%	11,900	0.5%	22,758	0.4%
−80	8675	0.3%	10,831	0.4%	19,506	0.4%
>80	12,056	0.2%	21,036	0.3%	33,092	0.3%
Total	232,539	0.5%	238,403	0.4%	470,942	0.4%

**Table 3:** Prevalence odds ratio of schizophrenia adjusted for sex, season of birth, and birth during an influenza pandemic.

Variable	N	%	OR (Crude)	IC 95%	OR (Adjusted *)	IC 95%
Age	470,942	0.44%	1.003	1.001–1.006	1.005	1.002–1.007
Sex						
Female	238,403	0.35%	1	-	1	-
Male	232,539	0.53%	1.506	1.380–1.645	1.516	1.388–1.655
Season						
Summer–Autumn	230,900	0.42%	1			
Winter–Spring	240,042	0.46%	1.115	1.023–1.216	1.112	1.020–1.212
Influenza pandemic						
No	400,203	0.42%	1	-	1	-
Any influenza Pandemic	70,739	0.56%	1.335	1.199–1.486	1.349	1.208–1.507
1957–1959 Pandemic	22,861	0.63%	1.515	1.278–1.796	1.476	1.244–1.750
1968–1970 Pandemic	28,780	0.52%	1.235	1.044–1.461	1.261	1.064–1.493
1977–1978 Pandemic	19,098	0.50%	1.203	1.013–1.430	1.280	1.072–1.528

* Adjusted for all variables in the model (age, sex, season, and influenza pandemic).

## Data Availability

The datasets generated for this study are not available due to the data protection law.
